# Surgery

**Published:** 1860-03

**Authors:** S. W. Gross

**Affiliations:** Lecturer on Surgical Anatomy and Operative Surgery, Philadelphia


### Bi-Monthly Abstracts;

OR, BI-MONTHLY NOVELTIES IN MEDICAL, PATHOLOGICAL, SURGICAL, OBSTETRICAL, PHYSIOLOGICAL, AND CHEMICAL SCIENCE.

### SURGERY.

BY S. W. GROSS, M.D.,

LECTURER ON SURGICAL ANATOMY AND OPERATIVE SURGERY, PHILADELPHIA.

I.—*Acupressure: A New Method of Arresting Surgical Hemorrhage.* By J. Y. SIMPSON, M.D., F.R.S.E., Professor of Medicine and Midwifery in the University of Edinburgh, etc. etc. (Edinburgh Medical Journal, January, 1860.)

IN a communication made to the Royal Society of Edinburgh, the distinguished author of this paper discusses at some length the effects produced by the application of silk ligatures to divided arteries, and insists upon the fact of their acting as setons in a wound and preventing union by the first intention. After reviewing the different improvements made to overcome the difficulty, he proposes to diminish the dangers of irritation, and to favor the chances of primary union by a new hemostatic process, by which no foreign body is left permanently in a wound.

To fulfill these indications he has devised what he terms acupressure, which he and other surgeons have successfully employed upon the human subject. It is made by means of slender needles of passive or non-oxydizable iron, which, in other respects, resemble the ordinary hare-lip pin. The needle is passed twice through the wound, and compresses, by its middle portion, the divided artery, a line or two on the cardiac side of the bleeding point. As soon as the vessel is supposed to be adequately closed, which will be, generally, on the second or third day, the instrument is removed and the adhesive process is thus not interfered with.

In introducing the pin the surgeon compresses the bleeding orifice with the tip of his left index finger, and with his right hand passes it through the cutaneous surface of the flap, until its point projects out on the raw surface of the wound, when it is carried across the artery, and brought out on the opposite side. If it be necessary to make more forcible pressure, a twisted suture may be cast around the instrument. The process may be better understood and imitated by the mode by which the stem of a flower is fastened to the lapel of a coat. A deep-seated artery may be compressed against a bone in the same manner.

The advantages of acupressure over ligation of arteries are stated by Professor Simpson in the following propositions:—

"1st. Acupressure will be found more easy, simple, and expeditious in its application than the ligature.

"2d. The needles in acupressure can scarcely be considered as foreign irritating bodies in the wound, and may always be entirely removed in two or three days, or as soon as the artery is considered closed; while the ligatures are true foreign irritating bodies, and cannot be removed till they have ulcerated through the tied vessels.

"3d. The ligature inevitably produces ulceration, suppuration, and gangrene at each arterial point at which it is applied; while the closure of arterial tubes by acupressure is not attended by any such severe and morbid consequences.

"4th. The chances, therefore, of the union of wounds by the first intention should be much greater under the arrestment of surgical hemorrhage by acupressure than by the ligature.

"5th. Phlebitis, pyæmia, etc., or, in other words, traumatic or surgical fever, seem not unfrequently to be excited by the unhealthy local suppurations and limited sloughings which are liable to be set up in wounds by the presence and irritation of the ligatures.

"6th. Such dangerous and fatal complications are less likely to be excited by the employment of acupressure, seeing the presence of a metallic needle has no such tendency to create local suppurations and sloughs in the wound, such as occur in the seats of arterial ligatures.

"And 7th. Hence, under the use of acupressure, we are entitled to expect both, first, that surgical wounds will heal more kindly and close more speedily; and, secondly, that surgical operations and injuries will be less frequently attended than at present by the disastrous effects and perils of surgical fever."

II.—*On the Treatment of Strangulated Hernia by the Taxis, and especially by forced and prolonged Taxis.* By DR. GOSSELIN, Surgeon to the Cochin Hospital, etc. (A paper read before the Paris Academy of Medicine, 26th of October, 1859. Gazette Hebdomadaire, Nos. 44 and 46, pp. 694 and 725, November 4th and 18th, 1859.)

AMUSSAT and Lisfranc long since advocated forcible and prolonged taxis in the reduction of strangulated hernia; but this treatment has not been generally recognized by surgeons as a safe procedure, preferring to employ it with as little force as possible, and not to prolong their efforts, in the majority of instances, over twenty minutes. During the last fifteen years, Dr. Gosselin has met with eighty-five cases of strangulated hernia, and has investigated very fully the treatment of which he is, at the present time, so warm a partisan.

In making the taxis, the surgeon should grasp the tumor in the usual manner, and commence his manipulations by making moderate pressure, acting, of course, upon that portion of the gut which was last protruded. If, in four or five minutes, the parts be not reduced, the pressure should be increased and both hands be used, at the same time that the surgeon throws, through his arms, the weight of his body upon the tumor. As the process is very fatiguing, it will become necessary, as happened in one-third of the author's cases, for an assistant to apply his hands also; thus constituting what Dr. Gosselin calls "the taxis with four hands." Or, if the operator be much fatigued, his place is supplied by a competent person. These efforts may be continued from thirty to sixty minutes, after which time, if the hernia is not returned, an operation will be called for.

Of the eighty-five cases of strangulated hernia, treated by the author in private and in hospital practice, only twenty-nine were subjected to the taxis; moderate force being sufficient in some instances to effect reduction, but in the great majority of cases the pressure was forcible, and continued usually about half an hour. Two of these patients died; the first being a case of femoral hernia, in which forced and prolonged taxis was employed unsuccessfully for three-quarters of an hour, and, as the patient would not consent to an operation, she died from the effects of the strangulation. In the second case, one also of femoral hernia, the fatal result may be attributed to the taxis.

The remaining twenty-seven patients all recovered without any accident, excepting one, who had slight symptoms of peritonitis, which, however, disappeared in five days. The duration of the strangulation in these cases dated, at the furthest, for inguinal hernia, three days; for femoral, thirty-six hours; and for umbilical, twenty-four hours—the form of hernia being, in seventeen inguinal, in seven femoral, and in three umbilical.

From these facts it would appear that inguinal hernias were not so firmly strangulated as other varieties; but when we compare the cases successfully reduced with those in which the taxis failed, we find that the failures are nearly as numerous as the successes; thus, Mr. Gosselin operated on sixteen patients with inguinal hernia, who had previously been subjected to the taxis by other surgeons, so that he is unable to say whether it had been made properly. In the majority of these cases the strangulation had existed more than forty-eight hours, so that he was fearful to return a gut which might have been gangrenous or perforated. He had but two cases of failure, in which the constriction had existed less than two days.

In regard to the adjuvants of the taxis in strangulated hernia, the author places no confidence in venesection, baths, poultices, purgatives, strychnia, coffee, and other measures, but very properly dwells upon the importance of position and the employment of chloroform in all cases. Without the latter agent it would be impossible to conduct the taxis properly, on account of the great pain which it occasions, and by no other means could the necessary muscular relaxation be obtained.

That femoral hernia proves most difficult of reduction is shown from the fact, that of thirteen cases in which forcible taxis was instituted, five were failures; whereas only two were unsuccessful in nineteen cases of inguinal hernia. Inasmuch as gangrene and perforation of the intestine take place much sooner in femoral than in inguinal hernia, the author would assign as the limit of time for the practice of forced taxis, in the former forty-eight hours, in the latter seventy-two hours.

III.—*A Case of Simultaneous Lateral Dislocation of both Knees.* By J. W. Hamilton, M.D., Professor of Surgery in the Starling Medical College. (Ohio Medical and Surgical Journal, January, 1860, p. 185.)

On account of the large size and strong ligamentous connections of the knee-joint, it is comparatively rare that the surgeon meets with a luxation of this articulation; and when it does take place laterally, whether externally or internally, the injury is most frequently incomplete. The case of Dr. Hamilton is, therefore, especially interesting, from the fact that both tibiæ were completely dislocated laterally and externally from the condyles of the thigh bones. The accident occurred to a German, forty-four years of age, of medium muscular development, who was bruised by the falling of a large pile of saw-dust. In addition to the dislocations, the right fibula was fractured just below its head, and the ligaments of the joints were so completely severed that the legs could be made to make almost any angle with the thighs. But slight manipulation was required to reduce the luxations and the integuments were unbroken.

The treatment consisted in keeping the limbs in an extended position, with extensive lateral support by means of folded comforters, and the continuous application of cold water, opium being administered internally. At the end of ten days active inflammatory symptoms having disappeared, the apparatus and cold water were discontinued, and slight, passive motion, with the external application of iodine, substituted.

On the forty-fifth day the motion of the knee-joints was perfectly free, but the right knee could be partially dislocated by exerting slight lateral pressure. The patient's limbs were bandaged and the knees supported with pasteboard splints, and he was allowed to walk about on crutches. A complete cure is eventually expected.

IV.—1. *Aneurism of the Radial Artery cured by digital compression.* By DR. DENUNCÉ, Adjunct-Professor of the Clinic of the St. Andrew's Hospital of Bordeaux. (Journal de Médicine de Bordeaux, December, 1859, p. 781.)

2. *Aneurism of the Crural Artery treated successfully by digital compression.* By M. RIBERI. (Gazetta Medica Italiana, and Edinburgh Medical Journal, November, 1859, p. 468.)

1. The first case occurred in the person of a mason, thirty-three years of age, who divided the lower portion of his left radial artery with a hatchet, the lips of the wound having been simply drawn together with lead plaster. On the tenth day after the accident hemorrhage took place, which was arrested by a compress and small splints, these being confined in their position by a bandage for three weeks.

When seen by Dr. Denuncé. seven weeks after the occurrence, the cicatrix was found to be firm. There was present a large, well-marked, false aneurism, the pulsations of which were completely abolished by pressure upon the artery above and below the tumor. The patient having been confided to the charge of two assistants, was placed recumbent, with the arm extended on a pillow and supported by a table, and the treatment was commenced at ten o'clock on the morning of the sixth of November, by instituting pressure with the fingers of both hands above and below the tumor. In six hours the tumor had become firmer and the pulsations were more feeble, and at nine o'clock in the evening, pulsation had entirely ceased. Digital compression was maintained until midnight, when a cork was confined over the artery by means of a roller. On the following morning, at nine o'clock, pulsation had not returned, but compression with the fingers was again established for nine hours, when the tumor was found to be hard, except at its superior portion, where there was some fluctuation, and the skin was very thin. Mechanical compression was continued for several days, with the effect of causing the tumor to decrease in size, and at no time was pain experienced. The patient was seen eleven months subsequently, when a small, hard point, about the size of a pea, marked the former site of the tumor.

2. M. Riberi instituted digital compression on the femoral artery as it passes over the horizontal ramus of the pubes, for a very painful aneurism of the lower third of this vessel. The patient was a young man who, several months previously, had fallen from a locomotive and severely concussed his spine, the accident being followed by the appearance of the aneurismal tumor and an attack of subacute myelitis. For the latter affection, as well as for syphilis, under which he labored at the time, he underwent treatment before the aneurism was attended to.

The pain and pulsation ceased after two hours' compression, and in four hours more the tumor became hard. Ice-water was now applied, and the aneurism gradually became firmer and smaller, and the treatment was discontinued at the lapse of four days.

V.—*Review of Cases of Stone in the Bladder.* By THOMAS P. TEALE, F.R.C.S., Surgeon to the Leeds General Infirmary. (Medical Times and Gazette, December 10, 1859, p. 571.)

Dr. Teale, in this interesting surgical contribution, places before the profession the statistics of the operations for stone. performed by him in private and in hospital practice from 1826 to 1859, and also notices the changes which he has made from time to time in his mode of operating.

The number of cases is eighty-seven, and they are classified under the following heads:—

| | Cases. | Recoveries. | Deaths. |
|---|---|---|---|
| 1. Lateral lithotomy in adults . . . . . | 35 | 22 | 13 |
| 2. Lateral lithotomy in children . . . . | 18 | 18 | – |
| 3. Lithotrity in adult males . . . . . | 15 | 14 | 1 |
| 4. Median lithotomy . . . . . . | 12 | 11 | 1 |
| 5. Stone in the female . . . . . . | 7 | 7 | – |
| | 87 | 72 | 15 |

The nature of the calculus is stated in sixty-one cases, from which we find that thirty-four were lithic acid; fourteen, phosphatic; eleven, oxalate of lime; one, lithate of ammonia; and one, cystic oxide. Fifty-seven of these cases were adult males, and the remaining thirty comprised twenty-three male children and seven females, whose ages ranged from four to sixty-three years.

From the above table it will be perceived that the lateral operation was performed, without a single death, on eighteen children, their ages varying from two and a half to thirteen years, and that all of the females subjected to an operation recovered. In four of the latter simple dilatation was performed; in two, superficial incision and dilatation; and in one, lithotomy. These statistics confirm the fact of the lateral operation being the most successful in subjects under the age of puberty.

The author discusses, more especially, the various operations for stone in the adult male, or patients after the age of puberty, and, from the table, we are surprised to see the mortality of the lateral operation, in which the only instruments used were the straight staff and knife of the late Mr. Aston Key. The operations were thirty-five in number with the result of twenty-two recoveries and thirteen deaths, the proportion of the latter being one in two and two-thirds of the cases.

Lithotrity was practiced in fifteen cases, of which fourteen recovered. The number of sittings in fourteen cases averaged six; the smallest number being two, the largest twenty-two. Dr. Teale prefers allowing the urine to distend the bladder in preference to injecting this organ with tepid water previous to the performance of the operation.

In seven of the adult males median lithotomy was performed, the result being one death and six recoveries. To these may be added five cases in which the operation was practiced on subjects under the age of puberty, all of whom recovered. In a note appended to his paper, the author states that the median process has been performed in Leeds twenty-three times, with the result of three proving fatal. Fifteen of these operations were performed on adults, two proving fatal.

We thus find, presenting the above facts in tabular form, that fifty-seven operations were performed by Dr. Teale on adult males with the following results:—

| Cases. | Recoveries. | Deaths. | Death. | Cases. |
|---|---|---|---|---|
| 35 Lateral lithotomy . . . . | 22 | 13 | or 1 in | $2\frac{2}{3}$ |
| 15 Lithotrity . . . . . | 14 | 1 | or 1 in | 15 |
| 7 Median lithotomy . . . . | 6 | 1 | or 1 in | 7 |

This table will serve to demonstrate the reasons why the author has changed his mode of operating from time to time, and he thus states the successive phases of his practice. 1. Lateral lithotomy in all cases. 2. Lateral lithotomy the rule, lithotrity the exception. 3. Lithotrity the rule, lateral operation the exception. 4. Median lithotomy the rule, lithotrity the exception.

For dilatation of the prostate, Dr. Teale advises the employment of the left index finger for children, whereas, in the adult, he uses a modification of Weiss's dilator for the female urethra. The instrument consists of three branches, with blades five inches and a half in length, and terminating in a slight bulb to prevent it slipping out of the bladder. The conjoint diameter of the instrument when closed, is three-fourths of an inch at its base, and half an inch at the bulb.

VI.—1. *A New Principle of Treatment and Apparatus for Vesico-Vaginal Fistula.* By ROBERT BATTEY, M.D., of Rome, Georgia. (Lancet, December 31st, 1859, p. 664.)

2. *On Vesico-Vaginal Fistula: Illustrating a New Method of Operation.* By ISAAC BAKER BROWN, ESQ., F.R.C.S. (Lancet, December 10th, 1859, p. 581.)

1. Dr. Battey's new apparatus for the treatment of vesico-vaginal fistula is a modification of Bozeman's button, and consists of a leaden shield, which, acting the part of a splint and compress, exerts moderate pressure upon the approximated edges of the fissure. For this principle alone the author claims originality, and thinks it possesses the advantages of producing a water-tight joint, impervious to the flow of urine; that it permits of more accurate apposition of the edges of the wound, thus favoring union by the first intention, and affords an opportunity of inspecting the lips of the fistule up to the time of their final closure.

When the tissues are rigid and there is great loss of substance, the apparatus, moreover, possesses the power of bringing down the vagina and uterus, so as to render the parts more accessible, thus admitting of their more easy closure, with less danger of the sutures cutting out.

The combined splint and compress is composed of a strip of sheet-lead, of convenient length, thick enough to be flexible, and usually five-sixteenths of an inch in width; one side of the bar is perforated with holes about three-sixteenths of an inch apart, and the other side is notched opposite the perforations. The edges of the fistule having been pared in the usual manner, and the stitches introduced, the apparatus is applied by passing the ends of the silver wires through the holes in the splint, and securing them by means of perforated shot, leaving about an inch of their ends free. Traction being made upon the sutures, the splint is drawn down upon the posterior edge of the fistule, and a strip of wood is passed under the wires and pressed upward, bringing the anterior lip in contact with the posterior, thus closing the opening. The splint is next turned down, and the proximal ends of the wires are pushed into the notches and the two ends are then twisted together, snipped off, and turned down on the splint.

In the *Southern Medical and Surgical Journal* for December, 1859, Dr. Battey reports a case successfully treated by his method, the apparatus being removed on the tenth day.

2. The new operation of Mr. Brown, which he has employed four times successfully, consists in substituting for the button a simple leaden bar clamp, slightly curved, and having a perforated nipple projecting from its back. The metallic sutures having been introduced, the two ends of the first wire are passed through the clamp, and held in the left hand, and the nipple of the clamp being seized with a pair of long forceps it is pressed back until the edges of the fistule are in apposition, when the nipple is firmly squeezed and the suture secured. The remaining sutures are treated in the same manner.

The author has succeeded in operating by his method within three-quarters of an hour in the worst cases, and therefore claims, as its advantages, its celerity, the certainty of perfect apposition of the edges of the fistule, its application to every fistule, no matter however tortuous, and the ease with which the suture is removed.

VII.—*Simultaneous Dislocation of both ends of the Clavicle.* By M. MOREL-LAVALLÉE. (Gazette des Hôpitaux, No. 33. 1859.)

M. Morel-Lavallée reports to the Surgical Society of Paris a case of this extremely rare injury, occurring in a man, forty-four years of age, who was compressed between the wheel of a carriage and a pile of wood. When seen by the author on the following day, the attitude of the patient was expressive of injury to the clavicle; and, on examination, the sternal extremity of the bone was found to be luxated forward, as denoted by the presence of a hard, circumscribed tumor just beneath the episternal fossa, and the other signs of this dislocation. The acromial end of the bone was placed in the middle of the space separating the side of the neck from the shoulder, and it projected underneath the skin, rendering its outline easily traceable. The clavicle appeared to have assumed an anterior-posterior direction, and the relaxed trapezius formed a small, globular, soft tumor. The supra and sub-clavicular fossæ were completely effaced, but the distance from the acromion to the sternum was the same on both sides. The sternal dislocation was reducible, and could be maintained in place by bandages, but the external extremity of the clavicle resisted all efforts at reduction.

VIII.—*The Value of Internal Incision in the Treatment of Obstinate Strictures of the Urethra.* By HENRY THOMPSON, F.R.C.S., M.B., etc. (Lancet, Amer. Edit., January, 1860, p. 25.)

In this paper, Dr. Thompson, after stating that he is a partisan of no particular mode of treatment for organic stricture, calls attention to the value of internal incision; believing, however, that dilatation is the safest and simplest method, and that Syme's operation is valuable in certain cases. The treatment by internal incision is as useful and desirable as either, but should only be resorted to when dilatation has proved useless or merely palliative. Incisions should be restricted, first, to strictures of very long standing, and so unyielding that dilatation produces no material good effect upon them; and, second, to those occurring in early life, and proving obstinate to dilatation; as in these cases the disease will recur, and the symptoms will become aggravated. Young and middle aged persons are more amenable to treatment, and the incisions should, therefore, be practiced in them to prevent future extensive disease of the urinary organs.

In the large proportion of the above cases internal urethrotomy affords a more efficient means of treatment than external incision, or in those subjects in whom the indurated tissue can easily be divided by a cut of moderate length. In no case is an instrument capable of making deep incisions to be employed, as the danger is much greater than by external division, when the stricture is of such an extent as to require a deep urethral incision necessary for its complete division. The narrowness of a stricture alone is not an indication for an operation, as it, usually, has not a corresponding relation to the intensity of the symptoms, but the strictures requiring incision are those characterized by non-dilatability and contractility; the presence or absence of these two physical characters, and not mere narrowness, are, therefore, to be considered in the application of any treatment.

The author lays down three rules for the successful practice of internal urethrotomy, namely, the cutting instrument must be passed completely through the contraction, and the incision be made from behind forward; the whole of the contracted part must be divided, and the lips of the wound must be held apart by the catheter to prevent primary union.

In the first condition, after having completely passed the stricture and cutting from behind forward, the incision is not jagged, as in the case where the urethrotome is passed from before backward, in doing which the folds of the mucous membrane of the urethra are carried in advance of the instrument, thus making an uneven cut, not altogether under the control of the operator. The latter mode is occasionally followed by scrotal or perineal abscess, which is very rare when the other method is employed.

To fulfill the second indication the limits of the stricture must first be accurately defined by the employment of an instrument with a bulbous extremity; and as there is always some tendency in the canal to contract in front and behind the stricture to the extent, usually, of a third of an inch, those portions must be included in the incision, or the disease will almost invariably recur at a sooner or later period. Subsequent catheterism with a large instrument, is positively essential for a radical cure.

Dr. Thompson still prefers Civiale's urethrotome for the division of strictures in front of the scrotum and in the bulbous portion of the urethra; but his own instrument admits of being made of smaller size than any previous one for cutting from behind forward, and is to be used when the stricture is so small that the urethrotome of Civiale cannot be introduced. It is very similar to the one employed by Mercier, of Paris, for incising the prostate, and consists of a canula terminating in a slightly-curved and bulbous extremity, which contains the blade of the instrument. In employing it, it is only necessary to withdraw the handle of the blade to the same extent as is done when the entire instrument of Civiale is used, the canula remaining *in sitû.* The degree of projection of the blade is not calculated to do any harm, and, when exposed, the distance from its point to the back of the canula equals the diameter of a No. 14 catheter.

In regard to hemorrhage, urinary extravasation, perineal abscess, and constitutional disturbance, which are apt to follow any operations upon the urethra, the author states that he has notes of forty-two cases in which internal incision had been practiced upon different portions of the canal without meeting with any accident, excepting in two instances, in which pretty free hemorrhage took place.

